# TEMPO-Oxidized Cellulose Nanofibril Films Incorporating Graphene Oxide Nanofillers

**DOI:** 10.3390/polym15122646

**Published:** 2023-06-11

**Authors:** Yoojin Kim, Young-Teck Kim, Xiyu Wang, Byungjin Min, Su-il Park

**Affiliations:** 1 Department of Sustainable Biomaterials, College of Natural Resources and Environment, Virginia Tech, Blacksburg, VA 24061, USA; 2 Department of Chemistry, College of Agriculture Environment & Nutrition Science, Tuskegee University, Tuskegee, AL 36088, USA; 3 Department of Packaging, Yonsei University, Wonju 26493, Republic of Korea

**Keywords:** nanofibrillated cellulose, TEMPO, graphene oxide, nanocomposite

## Abstract

To design a new system of novel TEMPO-oxidized cellulose nanofibrils (TOCNs)/graphene oxide (GO) composite, 2,2,6,6-tetramethylpiperidine-1-oxyl radical (TEMPO)-mediated oxidation was utilized. For the better dispersion of GO into the matrix of nanofibrillated cellulose (NFC), a unique process combining high-intensity homogenization and ultrasonication was adopted with varying degrees of oxidation and GO percent loadings (0.4 to 2.0 wt%). Despite the presence of carboxylate groups and GO, the X-ray diffraction test showed that the crystallinity of the bio-nanocomposite was not altered. In contrast, scanning electron microscopy showed a significant morphological difference in their layers. The thermal stability of the TOCN/GO composite shifted to a lower temperature upon oxidation, and dynamic mechanical analysis signified strong intermolecular interactions with the improvement in Young’s storage modulus and tensile strength. Fourier transform infrared spectroscopy was employed to observe the hydrogen bonds between GO and the cellulosic polymer matrix. The oxygen permeability of the TOCN/GO composite decreased, while the water vapor permeability was not significantly affected by the reinforcement with GO. Still, oxidation enhanced the barrier properties. Ultimately, the newly fabricated TOCN/GO composite through high-intensity homogenization and ultrasonification can be utilized in a wide range of life science applications, such as the biomaterial, food, packaging, and medical industries.

## 1. Introduction

Industries and manufacturers have recently started to substitute petroleum-based plastics with eco-friendly biocomposites for sustainable applications. Nanocomposites entirely derived from biomaterials have received attention due to their biodegradability, biocompatibility, and renewability, combined with their commercial viability. For example, the potential biopolymers to fulfill these desired benefits are: cellulosic plastics, starch plastics, soy-based plastics, and poly(lactic acid) (PLA) [1,2]. In terms of the environmental and ecological aspects, the most abundant biopolymer, cellulose, is becoming a promising material substituting traditional plastics. Nanofibrillated cellulose (NFC) is a good polymer matrix candidate with high stiffness and elastic moduli, making it suitable for the fabrication of nanocomposites [3,4]. Cellulose fibrils, ranging from 10 to 100 nm in width, are generally produced via two main processes: mechanical refining and high-pressure homogenization of natural pulps [5,6]. In spite of the high energy input by intense mechanical treatment, the complete isolation of cellulose fibril bundles is not attainable owing to the abundant hydrogen bonds tightly linking the fibrils [6]. To achieve further individualization of nanofibrils, a promising chemical disintegration method is using 2,2,6,6-tetramethylpiperidine-1-oxyl (TEMPO) regioselective oxidation by converting primary alcohols to aldehydes and carboxylate groups [7,8]. Sodium bromide (NaBr) is frequently used as a catalyst, and sodium hypochlorite (NaClO) is a primary oxidant for the in situ reaction of the TEMPO radicals under mild conditions [7,8]. The surface-modified cellulose becomes disintegrated by applying negative electrostatic repulsions and loosening the cohesiveness between microfibrils and ends up with transparent nanofibrils. TEMPO-mediated oxidation of cellulose introduces the carboxylate groups mostly at their surfaces. This phenomenon is attributed to the large crystalline regions and poor accessibility to the fibrils inside. Nevertheless, TEMPO-mediated oxidation yields a dense network of nanofibrils, providing a good distribution in water due to the abundant functional groups in certain hydrophilic portions. 

In recent years, the incorporation of nanofillers, such as organoclay, cellulose nanocrystals, carbon nanotubes (CNT), graphene oxide (GO), and polysilsesquioxane, has been widely used to attain the desired properties—mechanical and barrier properties [9,10]. Zhu et al. reported that TEMPO-mediated nanocellulose was effectively combined with GO to produce a unique network, driven by the morphology of the GO phase and stabilized by the intermolecular H-bonding between carboxyl groups and hydroxyl groups. However, this study did not show the effect of the mixing method on the physico-chemical and barrier properties of films [11]. Pottathara et al. investigated the effect of the composite structure of TEMPO-oxidized cellulose and GO on the dye-absorption properties of the film. However, this study also prepared the composite structure by means of a simple mixing method to produce a film-forming solution [12].

GO is composed of the sp^2^-hybridized carbon network and exists in a two-dimensional honeycomb structure. GO is one of the extensively used nanofillers in a polymer matrix because it shows excellent thermal, mechanical, and electrical properties. In addition, GO layers have abundant oxygen-containing functional groups—hydroxyl, epoxy, carbonyl, and carboxyl—and so they are prone to be dispersed homogeneously in an aqueous medium and cause intermolecular interactions between nanofillers and polymer matrices at a molecular level [13]. Due to the significant property enhancement even with a low GO content, it has been reported that nanocomposites with several polymer matrices, such as poly(vinyl alcohol) (PVA), poly(methyl methacrylate) (PMMA), and poly(bibenzimidazole), have been fabricated with graphene oxide in the past few years [13]. In order to satisfy the targeted properties, uniform dispersion of nanofillers in the polymer substrates is considered as a significant factor; however, the strong intra- and inter-molecular interactions of cellulose fibrils and van der Waals forces of GO itself might hinder the efficient intermolecular cohesiveness of two phases. Therefore, optimized and proper mechanical and chemical treatment should be applied for sufficient load transfer from the polymer matrices to graphene oxide [13]. In the present study, the polymer substrate is fabricated with nanofillers to construct a new system of novel hybrid nanocomposites. 

The objective of our research is (1) to disintegrate nanofibrillated cellulose to the greatest extent, in order to obtain the water-insoluble fractions after the TEMPO-mediated oxidation via centrifugation, and (2) to combine with graphene oxide for the reinforcement of the nanocomposite. It also includes the characterization of TOCN/GO composites using various analytical evaluations such as X-ray diffraction (XRD), Fourier transform infrared spectroscopy (FTIR), the water vapor transmission rate (WVTR), the oxygen transmission rate (OTR), dynamic mechanical analysis (DMA), field emission scanning electron microscopy (FESEM), thermogravimetric analysis (TGA), and tensile testing to understand various properties. The outcome of this research may be beneficial in fields requiring high barrier and mechanical performance, such as coating, composite reinforcement, packaging, and drug delivery; furthermore, it could be a potential nanomaterial as an adsorbent to remove organic pollutants and oils dispersed in water [14,15]. 

## 2. Materials and Methods

### 2.1. Materials

Never-dried nanofibrillated cellulose, made from northern bleached softwood kraft pulp, with ~3% consistency (dry pulp/slurry) and a width of 28.4 μm, was purchased from the University of Maine and used for further treatment. 2,2,6,6-Tetramethylpiperidine-1-oxyl (TEMPO, 98%, Sigma-Aldrich, Burlington, MA, USA), sodium hypochlorite (NaClO, 13%, Fisher Scientific, Waltham, MA, USA), sodium bromide (NaBr, 99+%, reagent grade, Sigma-Aldrich), hydrochloric acid (HCl, 0.1 N, Certified, Fisher Scientific), sodium hydroxide (NaOH, 0.1N, Certified, Fisher Scientific), and ethanol (C_2_H_6_O, 95%, Decon Labs, King of Prussia, PA, USA) were used as received. Graphite (particle size of <20 μm) and microcrystalline cellulose (MCC) powder were obtained from Sigma Aldrich, USA.

#### 2.1.1. Preparation of Exfoliated Graphene Oxide

A modified Hummers method was used to prepare exfoliated graphene oxide from graphite [16]. In this process, 10 g of graphite powder was added to 250 mL of sulfuric acid (H_2_SO_4_) in a flask at room temperature. The mixture was placed in an ice bath followed by the addition of 35 g of potassium permanganate (KMnO_4_), which was then allowed to react at 35 °C for 12 h. The solution was cooled to <5 °C in an ice bath again. Excess hydrogen peroxide (H_2_O_2_) was gradually added to the flask with stirring to reduce unreacted KMnO_4_. Once the mixture turned bright yellow and bubbles were observed, it was washed repeatedly with distilled water until a pH of 7 was achieved. The resulting mixture was transferred to a conical centrifuge tube and centrifuged until no more supernatant was observed. Lastly, liquid nitrogen was utilized to freeze the neutralized graphene oxide, which was subsequently obtained in powder form via freeze drying. The exfoliated graphene oxide was characterized by XRD and stored in a vacuum desiccator.

#### 2.1.2. TEMPO-Mediated Oxidation of Cellulose

Nanofibrillated cellulose (20 g) was suspended in distilled water (2 L) using a domestic blender. Then, 0.25 g of TEMPO and 2.5 g of sodium bromide (1X TEMPO) were added to a 3000 mL three-neck round bottom flask, and the mixture was agitated with an overhead stirrer at 300 rpm. For 0.5X TEMPO and 2X TEMPO, the amount of TEMPO and NaBr was reduced by half (0.125 g, TEMPO and 1.125 g NaBr) and doubled (0.5 g, TEMPO and 5 g, NaBr), respectively. Then, 46 mL of 13% sodium hypochlorite (NaClO, 4.459 mmol/g cellulose) was gradually added to the cellulose slurry with a syringe pump at a rate of 1500 μL/min at room temperature. The pH of the solution was maintained at 10 ± 2 via the drop-wise addition of 0.5 N NaOH. Once no further change in pH was detected, 150 mL of ethanol was added to quench the oxidation reaction completely. The oxidized cellulose was washed thoroughly with distilled water by means of centrifugation at 5000 rpm for 30 min at 0–5 °C. This process was repeated several times until the pH of the cellulose sediment after the washes was adjusted to 7. The TEMPO-oxidized cellulose was stored at 4 °C for further treatment. The TEMPO-oxidized cellulose nanocomposites prepared at different oxidation levels (0.5X, 1X, and 2X TEMPO) were denoted as 0.5X, 1X, and 2X TOCN, respectively.

#### 2.1.3. Preparation of TOCN/GO Nanocomposites

**Figure 1:**
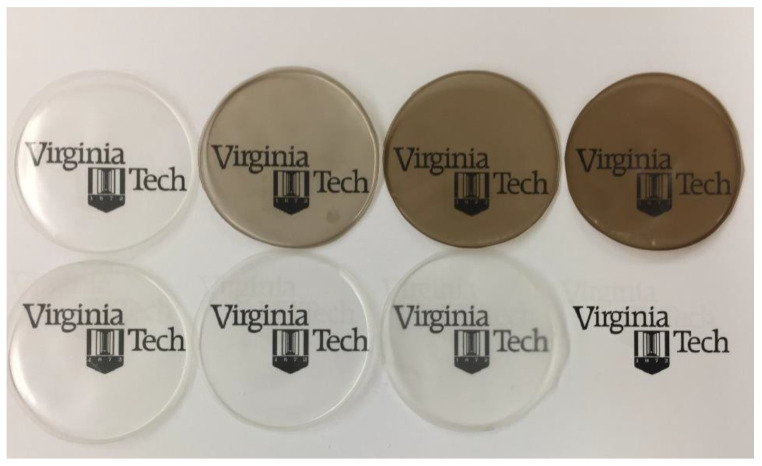
The TOCN/GO films produced in this study. Note: The top line shows Neat NCF, 0.4, 1.2, and 2.0%, respectively. The bottom line depicts 0.5X TOCN, 1X TOCN, and 2X TOCN, respectively.

TEMPO-oxidized cellulose was mixed with GO with a high-pressure laboratory homogenizer (MINI DeBEE). The TOCN/GO solution of the different concentrations were continuously agitated and homogenized five times using a 200 μm nozzle. The TEMPO-oxidized cellulose nanocomposites were prepared with the addition of various graphene oxide percent loadings: 0.4, 1.2, and 2.0 wt% with respect to dried cellulose, which were denoted as TOCN/GO-0.4, TOCN/GO-1.2, and TOCN/GO-2.0, respectively. The MCC/GO nanocomposite was also prepared as a reference and denoted as MCC/GO-2.0. The water content of the TEMPO-GO dispersions was evaluated in a moisture analyzer (OHAUS model MB45) in triplicate. Nanocomposite films were then cast from the cellulose/GO suspensions on a Petri dish at 50% RH and room temperature. The films were prepared as shown in Figure 1.

### 2.2. Characterization

#### 2.2.1. Determination of Carboxylate and Aldehyde Contents

The electric conductivity titration method verified the carboxylate content of the TEMPO-oxidized cellulose. A dried cellulose sample (0.4 g) was mixed with 0.1 N hydrochloric acid (100 mL) for an hour to protonate all of the carboxyl groups. Then, 0.1 N NaOH (95 mL) was added, and the solution was adjusted to 300 mL with deionized water. The mixture was then titrated with 0.1 N NaOH solution. Carboxylate contents were determined on a Metrohm 905 Titrando and calculated from Equation (1)

(1) $$\mathrm{carboxylate}\;\mathrm{groups}\;(\mathrm{mmol}/g)=\frac{c\;\times \;v}{m}=\frac{c\left(v_{2}-v_{1}\right)}{m}$$

where *c* is the molar concentration of NaOH (in M), *m* is the mass of the solid cellulose sample (in g), and *v* is the difference between the volume of NaOH (in mL) consumed to neutralize the excess hydrochloric acid and carboxylic acid [17]. The measurements were repeated in triplicate.

#### 2.2.2. Fourier Transform Infrared Spectrometry (FT-IR)

FTIR spectra of TOCN-GO composite films were recorded on a Nicolet 8700 FT-IR Spectrometer (Thermo Scientific, Waltham, MA, USA) using the attenuated total reflection (ATR) method. The data were collected from 4000 to 500 cm^−1^ with 64 scans at a resolution of 4 cm^−1^. A background spectrum was also collected before each sample.

#### 2.2.3. X-ray Diffraction Analysis (XRD)

The crystalline morphology of TOCN/GO nanocomposite was analyzed on a Bruker D8 XRD at room temperature using Cu-Ka (wavelength, 0.15406 nm) radiation conducted at 40 kV and 40 mA. The diffraction data were collected from the 2ϴ range of 5 to 60° with a scanning rate of 4°/min. The baseline correction was automatically applied for a better comparison, and the percent crystallinity ($\%\chi_{c}$) of each sample was calculated via the following equation:

(2) $$\chi_{c}\left(\%\right)=\frac{Total\;area\;of\;crystlline\;peaks}{Total\;area\;of\;all\;peaks}\;\times \;100\%$$

#### 2.2.4. Dynamic Mechanical Analysis (DMA)

Dynamic mechanical analysis was carried out on a Q800 DMA in tension mode. Specimens with 20 ± 0.5 mm length and 5.0 ± 0.1 mm width were used. The storage modulus (G’) and the loss modulus (G”) were investigated as a function of increasing temperature, ranging from 0 to 150 °C at a heating rate of 5 °C/min. A preload force and amplitude of 0.01 N and 20 μm, respectively, were used during the test.

#### 2.2.5. Mechanical Testing

The mechanical properties, such as the tensile strength and Young’s modulus, were determined on a TA-XT 32 texture analyzer from Texture Technologies. Specimens with at least 50 mm length and 15 mm width were measured at 6 mm min^−1^ and a 10 mm gauge length under ambient temperature conditions. Five replicates for each specimen were measured and normalized by thickness, and their average value was calculated. All the films were stored in a humidity-controlled chamber at RH 50% for 48 h before each use.

#### 2.2.6. Morphological Analysis

The field emission scanning electron microscopy (FESEM) was carried out on a LEO (Zeiss, Oberkochen, Germany) 1550 operated with an acceleration voltage of 2 kV. The cross-section of the sample was obtained by fracturing it in liquid nitrogen to assess the cross-sectional morphology. The TOCN/GO nanocomposite films were coated by sputtering with a thin layer of iridium.

#### 2.2.7. Water Permeation Analysis

The water vapor transmission rate (WVTR) was determined to measure the volume of water vapor diffusing through a film per unit area and time using the i-Hydro 7500 water vapor transmission rate testing system (Labthink International, Inc., Medford, MA, USA). All the specimens were conditioned at 50% relative humidity before each test. The edges of the specimens were masked using aluminum foil tape (average thickness of 30 ± 3 μm and test dimension of 2.5 × 2.5 cm). The WVTR of TOCN/GO nanocomposite films were measured in triplicate and a standard mode (4 cycles) at 50% RH and 23.5 °C. The flow rate of the nitrogen purge and humidified gas for each cell was adjusted to 100 V ± 2 mL/min. The WVTR was normalized by the partial pressure difference and thickness to obtain the water permeation coefficient. 

#### 2.2.8. Gas Permeation Analysis

The oxygen transmission rate (OTR) of TOCN and GO composite were determined using an oxygen analyzer (OxySense 5250i, Dallas, TX, USA) under ambient temperature conditions with 0% relative humidity. The gas permeation chamber was divided into two wells by the sample film. Pure nitrogen (a carrier gas) and oxygen (a test gas) were purged into the sensing well and the driving well with a flow rate of 1 mL/min. At least three replicates for each specimen masked using aluminum foil tape (average thickness of 30 ± 3 μm and test dimension of 38 × 38 mm^2^) were measured. The OTR was collected every five minutes and stopped once the oxygen concentration reached a steady state, with a regression coefficient of 0.99 or higher. Each sample was preconditioned in a vacuum oven at the desired 50% RH for 48 h.

#### 2.2.9. Thermal Gravimetric Analysis (TGA)

TGA was conducted on a TA Q500 TGA to observe the thermal degradation behavior of the TOCN-GO nanocomposites as the effect of the oxidation state and GO content. Approximately 10 mg of each sample was heated from room temperature to 700 °C at a rate of 10 °C/min under nitrogen.

#### 2.2.10. Statistical Evaluation

All data were analyzed by means of one-way analysis of variance (ANOVA) to determine the difference between samples, TEMPO treatments, and GO concentration. Minitab Software model 14.12.0 (Minitab Inc., State College, PA, USA) was used. Tukey’s test was used to carry out the difference of means between pairs with a 95% confidence interval.

## 3. Results and Discussion

### 3.1. Surface Charge of Neat NFC and TOCN/GO Nanocomposites

The C6 primary hydroxyl groups were converted to carboxylate groups via NaClO/NaBr/TEMPO oxidation at three different TEMPO and NaBr catalyst levels [5]. This was measured via conductometric titration based on the consumed amounts of NaOH. As shown in Table 1, the charge densities of neat NFC, 0.5X, 1X, 2X TOCN, related to carboxyls, were 0.183, 0.280, 0.370, and 0.425 mmol per gram of cellulose, respectively. In the presence of an excess of NaClO, the amounts of TEMPO/NaBr catalysts are quite proportional to that of the converted C6 carboxyls of cellulose, providing a correlation coefficient R^2^ = 0.91.

### 3.2. Dispersion of GO Fillers in a TOCN Matrix

**Figure 2:**
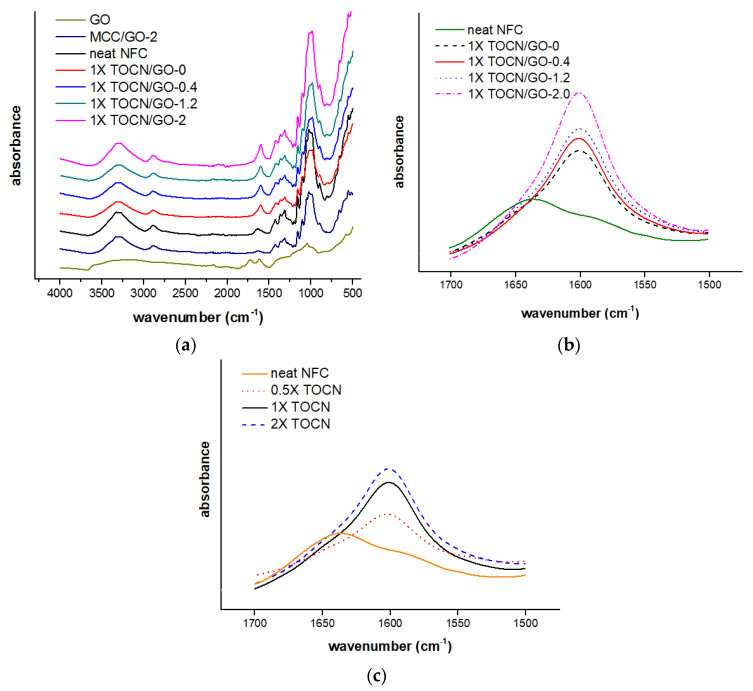
Stacked FTIR spectra of neat NFC and TOCN/GO composites (**a**) and FTIR absorption spectra of 1X TOCN/GO with various GO loadings (**b**) and TOCN films with different degrees of oxidation (**c**) at 1600 cm^−1^.

In the present study, FTIR was used to investigate the presence of graphene oxide and to determine the degree of oxidation by comparing the peak intensities. The FTIR spectra of neat NFC, neat GO, and TOCN-GO composites with various GO contents are displayed in Figure 2a. It is evident that the broad and strong peaks centered at 3330 and 2890 cm^−1^ are relevant to the stretching of the hydroxyl groups and carboxylate groups, respectively. The broad region corresponding to carboxylate groups also overlaps with the absorption of C-H stretching [18,19]. In addition, the characteristic absorption at 1630 cm^−1^ is indicative of stretching vibrations of carboxylate groups. This result is quite in accordance with that reported by Mandal et al. [20]. In their study, the spectrum of carboxymethyl cellulose showed intense peaks at 2917 and 1631 cm^−1^, corresponding to the stretching vibration of C-H and carboxylate groups. Other fingerprint absorptions of −CH_2_ scissoring, −OH bending, and C–O–C pyranose ring vibration are detected at 1430, 1373, and 1030 m^−1^, respectively [21]. As presented in Figure 2a, the FTIR spectrum of GO represents a broad peak at 3183 cm^−1^, which corresponds to the hydroxyl groups, and the peaks at 1729 and 1616 are associated with C=O and C=C stretching vibrations, respectively [22]. The peak assigned for C=O stretching vibration of GO, in general, should be detected around 1730 cm^−1^, but it overlapped with the carboxylate moieties region. Additionally, most of the spectral features of TOCN/GO composites are analogous, except for the intensity of the peaks, as shown in Figure 2b,c. For example, the higher absorbance at 1630 cm^−1^ in the spectra confirms the increased GO loadings in TOCN/GO nanocomposites due to the introduction of more oxygen-containing groups. In addition, this study found that the peak at 1630 cm^−1^ showed higher intensities as the degree of oxidation became more apparent for 2X TOCN. This is because the amount of carboxylate moieties converted from the C6 primary hydroxyls of cellulose increases in accordance with the amount of TEMPO involved in the reaction. This observation complies with the characteristic absorption spectra obtained by Shao et al. [23]. 

### 3.3. Crystallinity of the TOCN/GO Nanocomposites

**Figure 3:**
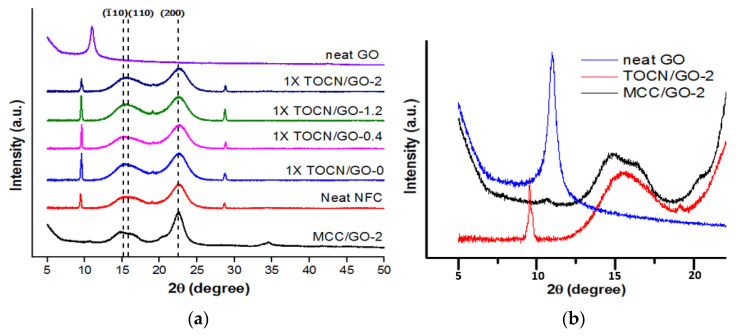
XRD patterns of neat NFC, GO, and TOCN/GO composites (**a**) and enlarged view of the XRD peak region of GO (**b**).

X-ray diffraction analysis was utilized to investigate the change in the degree of crystallinity upon the TEMPO oxidation and the inclusion of graphene oxide. As shown in Figure 3, the X-ray diffraction patterns of pristine NFC represent three characteristic peaks at 2ϴ = 15.4°, 16.6°, and 22.7°, assigned to the ($\underline{1}10$), (110), and (200) planes of the crystalline form of cellulose I, respectively [15]. The sharp intense peaks around 9.5°, 19.1°, and 28.7° were also observed from the XRD spectra of pristine NFC films, which were assumed to be impurities of the as-received untreated nanocellulose. According to ^13^C NMR and XRD spectra of the TEMPO-oxidized cotton linters [7], the carboxylate and aldehyde groups are present mainly at the C6 position of the cellulose surface and in non-crystalline regions of microfibrillated cellulose [7]. Correspondingly, Figure 3a illustrates that the characteristic intense peaks of all XRD profiles are very similar to each other, although the width of each peak is slightly widened compared to MCC/GO-2. This indicates that neither TEMPO-mediated oxidation nor the incorporation of GO highly affects the crystalline form. With respect to the crystallinity, the crystallinity of the native celluloses ranges from 59 to 95% depending on cellulose source materials [24,25,26]. 

Table 2 displays that the percentage of crystallinity derived from the ratio of the area of all crystalline peaks to the total area was not significantly changed even with various amounts of TEMPO and the addition of GO, as expected. For example, in the study from Saito et al., the percent crystallinity of neat spruce holocellulose was 59%, which was analogous to that of TEMPO-oxidized spruce holocellulose (60%) [25]. Our result agrees with those of Saito et al. Despite the oxidation with 10 mmol/g cellulose of NaClO, a relatively high amount, the crystallinity of cellulose and crystal size of cellulose I were almost identical [7]. Although it is conjectured that the increased amounts of carboxylate and aldehyde on the surface of cellulose I crystallites might attribute to this slight increase, this result substantiates that the TEMPO-mediated oxidation only occurs either on cellulose crystal surfaces or in the amorphous regions without any chemical modification inside of cellulose I crystallites.

In general, the exfoliation of graphene oxide is more facilitated compared to graphene and graphite in the polymer matrix. However, the strong van der Waals interactions might still cause restacking, agglomeration, and heterogeneous dispersion even with advanced mixing, especially when the number of nanoparticles reaches the critical loading [27]. Nevertheless, the well-distributed GO nanofillers were confirmed by XRD analysis. The homogeneous dispersion state can be proven by the absence of the (001) plane of graphene oxide, corresponding to the sharp peak observed at approximately 10.9° [28]. As shown in Figure 3b, the XRD patterns of MCC (microcrystalline cellulose), which is physically mixed with 2% GO without further treatment, showed a small GO peak. This indicates that the GO nanoparticles were not successfully intercalated into the MCC matrix with physical mixing itself. Meanwhile, the peak around 10.9° was not observed in the XRD patterns of TOCN/GO-2. This phenomenon signifies that the mechanical and chemical TEMPO treatments applied here in the study played a significant role in the exfoliation or complete separation of the graphene oxide sheets in the polymer matrix, not yielding the diffraction peak at 10.9°. 

### 3.4. Viscoelastic Properties of the TOCN/GO Nanocomposites

**Figure 4:**
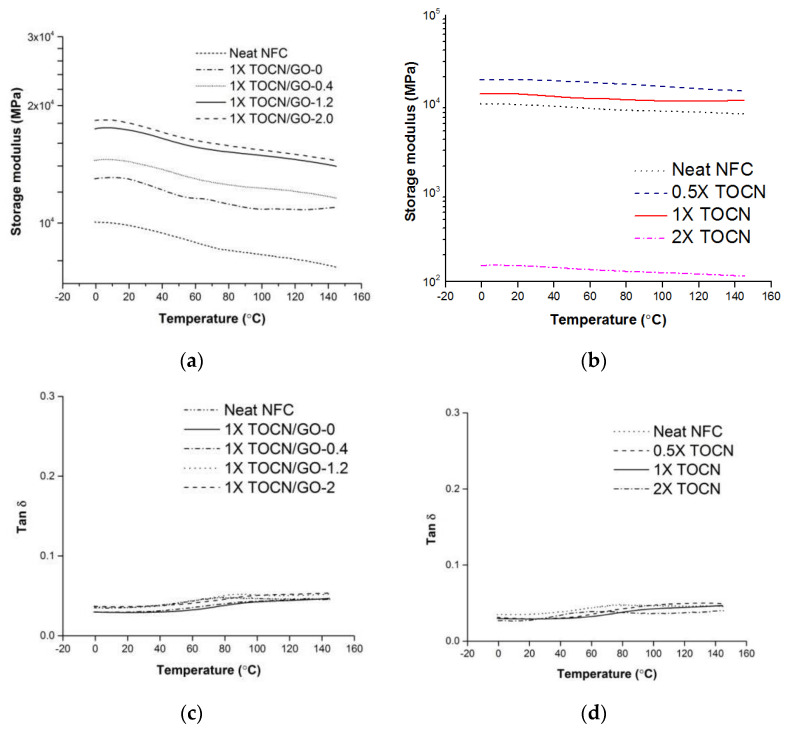
Storage moduli of the TOCN/GO composite samples with different contents of graphene oxide (GO) (**a**) and different degrees of oxidation (**b**). Tan δ of the TOCN films with different contents of graphene oxide (GO) (**c**) and different degrees of oxidation (**d**).

Figure 4a displays the logarithm of the storage modulus values as a function of temperature ranging from 0 to 150 °C. The storage modulus increased with increasing GO loadings; however, it remained comparatively independent of temperature, ascribed to the high degree of crystallinity and hydrogen bonds [29,30]. Bulota et al. also addressed that the storage modulus of neat cellulose was not strongly affected by a temperature increase, resulting in a slight decrease in storage modulus only [29].

The 1X TOCN/GO-2 showed the highest storage modulus over the whole temperature range. The enhancement of the storage modulus is caused by the effective load transfer from the TEMPO-oxidized cellulose matrix to GO nanofillers. The storage modulus of TOCN/GO nanocomposites with loadings of 0, 0.4, 1.2, and 2% at 23 °C correspond to percentage increases of 13%, 25%, 51%, and 57%, respectively, compared to neat NFC. As for the effect of the degree of oxidation, the TEMPO oxidation caused an increase in the storage modulus in accordance with the results obtained from Johnson et al. [9]. The increase in the storage modulus with the increasing oxidation level can be explained by the stronger fibrous network and entanglements of the highly separated fibril, limiting the segmental backbone motion of the cellulose chains around the nanofillers [31,32,33]. The dynamic moduli of 0.5X and 1X TOCN films, compared to neat NFC, created a stronger TOCN structure when there is an optimal concentration of TEMPO and NaBr for oxidation. The storage modulus of 1X TOCN was increased by 12% compared to neat NFC. Surprisingly, the 2XTOCN film exhibited an unexpectedly low storage modulus. A 2X TOCN film represented an approximately 100-fold decrease in storage modulus at 23 °C due to the increased void volume by severe oxidation. Despite the large number of interconnections in the network between fibrils, as predicted, the void volume generated on the surface was dominant in this case. 

Tan δ (E”/E’), also called a damping factor, indicates the dissipated energy of a polymer. However, peak broadening of TOCN/GO nanocomposites and a shift of the tan δ peaks were not detected as shown in Figure 4. The change in tan δ of TOCN upon heating is not significant, as reported by Bulota et al. [29]. 

### 3.5. Mechanical Properties

**Figure 5:**
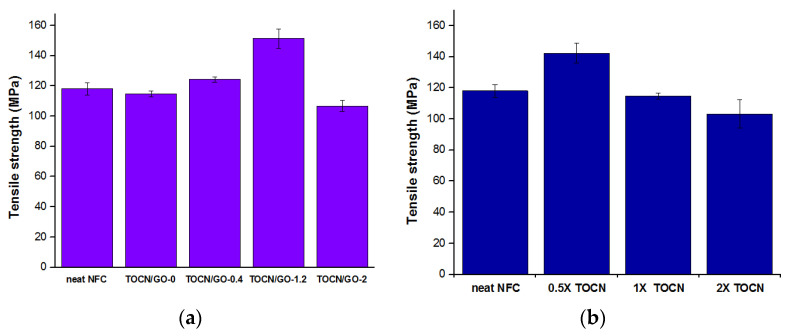
Tensile strength of neat NFC and 1X TOCN/GO composite films with different content of graphene oxide (GO) (**a**) and different degrees of oxidation (**b**).

The improvement in the mechanical properties of the nanocomposites is determined by the dispersion state, the degree of exfoliation of GO, and the interfacial interaction between the polymer matrix and nanofillers (Figure 5 and Table 3). Wang et al. found that the tensile strength of regenerated MCC (microcrystalline cellulose) composite films with the addition of 0.5 wt% of GO increased by 64.7% [14]. However, the mechanical properties of regenerated MCC-GO composites were worse than those of the neat MCC film with a GO content over 1 wt% due to the inhomogeneous dispersion of GO in the polymer matrix. Figure 5a shows the average tensile strength values. In addition, Young’s modulus increased from 1.1 GPa for neat NFC to 1.3, 1.5, 1.6, and 2.1 GPa for TOCN/GO-0, 0.4, 1.2, and 2.0, respectively. Based on our study, the tensile strength of TOCN films consistently increases as the percent loadings of GO increase, except for the 2 wt% GO addition (107 MPa). The decreased mechanical performance of TOCN/GO-2 can be explained by the decreased intermolecular cohesiveness between the polymer matrix and reinforcing phases, since it went beyond the greatest load, causing aggregation.

Han and Feng et al. reported that the regenerated cellulose composite films with a high concentration (5%) of GO improved the tensile strength by forming hydrogen bonds [34,35,36]; on the contrary, the composite films with high loadings of GO studied by Liu et al. showed a reduction in the mechanical properties when they went beyond a critical loading (0.5%), which was caused by a poor distribution state and the restacking of graphene oxide layers [34]. Figure 5b represents the different tensile strength tendencies according to the degree of oxidation. The tensile strength of 0.5X TOCN film is superior to that of other films, and it tends to decrease as the oxidation level increases. As analyzed by the DMA, 2X TOCN indicates a drastic worsening in mechanical properties since the increased number of hydrogen bonds between TOCNs and GO was not enough to offset the void volume. This result is also correlated with the SEM analysis.

### 3.6. Morphology of TOCN.GO Nanocomposite Films

**Figure 6:**
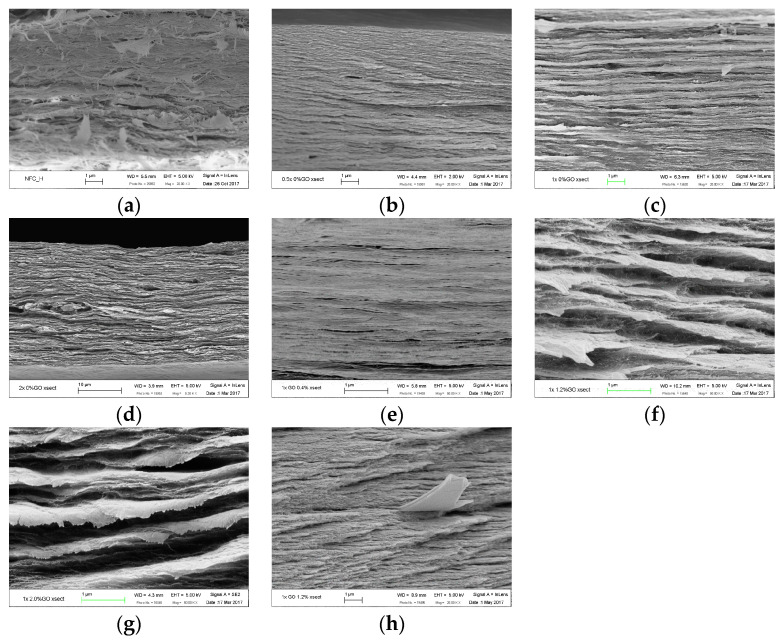
The cross-sectional SEM images of neat NFC (**a**), 0.5X TOCN (**b**), 1X TOCN/GO-0 (**c**), 2X TOCN (**d**), 1X TOCN/GO-0.4 (**e**), 1X TOCN/GO-1.2 (**f**), 1X TOCN/GO-2 (**g**), and the protruded graphene oxide sheets (**h**).

A FE-SEM (field emission scanning electron microscopy) was used to investigate the uniformity of fibril layers and any morphological changes upon the different degrees of oxidation and GO loadings. According to several studies, it has been found that surface modification via TEMPO does not influence the morphology of any TOCN samples [5]. Even with the introduction of the high amount of carboxylate groups, the original cellulose structure tends to be sustained since the oxidation preferably occurs on the surfaces and amorphous disordered regions [25]. However, other studies reported some morphological changes after oxidation. The commonly observed layered structure of randomly assembled fibers (Figure 6b–g) can result from the disintegrated fibril interactions, followed by the formation of hydrogen bonds among adjacent fibrils in a parallel direction, creating a layered structure. The layers are piled up on each other via interlayer van der Waals forces in the a–b plane of the unit cell, which is perpendicular to the chain axis [37,38].

The SEM images of neat NFC still demonstrate randomly oriented fibril bundles in a less compact form (Figure 6a). In contrast, cross-sectional SEM images of TOCN nanocomposite films (Figure 6b–g) revealed a considerably fibrous and dense layered structure composed of thin individual fibril layers. In our study, all TOCN films display highly interconnected and compact cellulose layers compared to neat NFC, but 2X TOCN has relatively more significant and less dense layers, along with an increased void volume caused by extreme oxidation [39].

Furthermore, with the incorporation of GO, the TOCN/GO films show homogeneity from the interior to the surface, which might lead to good adhesion between nanofillers and the polymer matrix. As a result, GO sheets can increase the reinforcement ability in the nanocomposites and successfully transfer the load from the TOCN matrix to GO unless it goes beyond the critical loading. Meanwhile, since the well-exfoliated GO layers were intercalated into the TOCN matrix rather than being stacked on top of each other, they were rarely found in the SEM images, as presented in Figure 6h. Han et al. reported that the apparent grooves created by fracturing the films were ascribed to uneven stiffness and the composite network between GO and chitosan, which has a similar backbone structure [40]. In our work, as the contents of GO increase, especially in 1X TOCN/GO-2, a more obvious stratification phenomenon can be observed. 

### 3.7. Gas Permeation Properties of Neat NFC and TOCN/GO Nanocomposites

Water vapor and oxygen barrier properties play significant roles in food packaging, since food decay is vulnerable to these two factors. The water vapor transmission rate (WVTR) is measured according to the amount of water vapor passing through the film per time and unit area until it reaches a steady state. Several physico-chemical parameters influence the WVTR of films, such as chemical composition, orientation, polarity, crystallinity, and void volume. Typically, a higher degree of crystallinity impedes the diffusion of water vapor molecules through the impermeable crystalline regions due to a more organized and dense network [41,42]. However, there was no significant difference in crystallinity, as observed in XRD, so the change in barrier permeability can be explained by other factors, such as the void volume, tortuous pathways, and a strong fibrous network formed by intermolecular interactions between the oxidized cellulose and nanofillers. A series of water and gas permeation tests on TOCN/GO nanocomposites were carried out, and the results are presented in Table 3 and Table 4. The thickness of the film is one of the parameters affecting the transmission rate by limiting the diffusion of oxygen and water molecules through the films. All of our samples showed statistically the same thickness (52 µm). For comparison purposes, the permeability of the films was reported [43,44].

The cellulosic materials tend to show higher values in the WVP compared that of the conventionally used packaging material, low density polyethylene (LDPE) (9.14 × 10^−15^ g·cm/cm^2^·s·Pa) [45]. Their WVPs remained higher because the hydrophilic cellulosic materials have a higher water affinity of their surfaces; additionally, the severe oxidation led to the cellulosic network structure being more loosened by the negative electrostatic repulsion [43].

As shown in Table 3, all of the WVPs among the different concentrations of GO were not significantly different from each other. However, the WVPs decreased after the moderate TEMPO-mediated oxidation, such as 0.5X and 1X TOCN, and increased again at 2X TOCN. This might result from an increased surface nanoporosity from the remarkably liberated fibrils when the extreme oxidation with the highest concentration of catalysts was applied. 

Although the water vapor barrier properties were still insufficient overall due to the hygroscopic nature of NFC, cellulosic materials generally tend to exhibit good oxygen barrier properties since the polar polysaccharides hinder the permeation of non-polar oxygen molecules due to repulsion.

All OTR values of TOCN films measured at 0% RH are higher than that of neat NFC. The TEMPO oxidation formed a highly packed cellulose network generated by fibrils with smaller dimensions, as observed in the SEM images. With respect to the effect of GO, the incorporated GO sheets enhanced the barrier properties by forming tortuous pathways against the oxygen molecules and retarding the diffusion of permeable gas molecules through the TOCN matrix. Meanwhile, the transmission rate of non-polar oxygen molecules might be influenced by the polarity of the film, expecting an increase in OTR since the C/O ratio in NFC is roughly 1.2, and the C/O ratio of GO is close to 2. However, the effect of the increased tortuosity and intermolecular interaction between the negatively charged TOCNs and GO was greater than that of the slight reduction in polarity after the addition of GO, resulting in a decrease in OTR values. In this study, the oxygen permeability of the TOCN film with 1.2% GO showed the lowest value at 175 mL/m^2^/day, which was even lower than that of the 2% GO-added film. Therefore, the 1.2% GO addition to the cellulose matrix is determined to be an optimal concentration for improving the barrier properties. As for the effect of the degree of oxidation, the higher oxidation level (1X TOCN) compared to 0.5X TOCN induced a decrease in the OTR from 669 mL/m^2^/day to 176 mL/m^2^/day. The OTR of 2X TOCN was expected to show the highest OTR value, but the consistent oxygen gas flow over time was not measurable. The reduced entanglement with shorter lengths of fibrils may have an influence on the OTRs of the TOCN films since the severe oxidation treatment tends to lower the degrees of polymerization of TOCN fibrils. Therefore, its exceptionally increased surface area, that is, void volume, occurred due to the less entanglement of the fibrils. It is concluded that the WVPs are still high, whereas the oxygen barrier properties were enhanced after the treatments.

### 3.8. Effect of TEMPO Oxidation on Thermal Properties of the Nanocomposites

**Figure 7:**
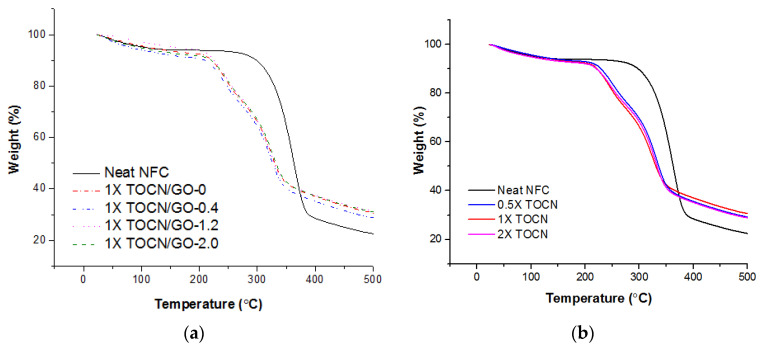
TGA curves for neat NFC and TOCN/GO composites at different GO loadings (**a**) and degrees of oxidation (**b**).

Figure 7 presents the onset temperature of the thermal degradation of TOCN-GO nanocomposites with different GO loadings. The TGA profiles of TOCN-GO nanocomposites exhibit three remarkable weight loss transitions. The first zone below 100 °C is attributed to the loss of moisture [21]. The significant weight loss observed in the range of 217–338 °C is due to the release of the oxygen-containing functional groups of thermally unstable graphene oxide, such as hydroxyl and epoxy, and the decarboxylation of TOCNs [21]. The last degradation region, above ~480 °C, is assigned to the degradation of graphene oxide in the composites. 

All TOCN films after oxidation demonstrate a distinctive effect on thermal degradation temperature. The TEMPO treatment was effective in promoting thermal degradation owing to the decarboxylation of hydro-glucuronate units via carbanion formation upon heating [46]. Fukuzumi et al. also reported that the onset of the thermal degradation of NFC with surface modification via TEMPO-mediated oxidation shifted to a lower-temperature region [47]. Pristine NFC was about 100 °C higher than that of TEMPO-treated NFC [4]. This corresponds to our TGA results. As for TEMPO treated 1X TOCN/GO-0, the thermal degradation rate was noticeably increased compared to the original NFC in Figure 7. The degradation onset temperature of the oxygen-containing functional groups decreased by 100 **°**C. This could be explained by the fact that the larger surface area accompanied by the chemical and mechanical disintegration processes promoted the more exposed portions of cellulose, leading to higher weight loss per unit of the rise in temperature [43]. However, the TGA curves for TOCNs with carboxylate groups of 0.280, 0.370, and 0.425 mmol/g were undistinguishable, and the addition of GO does not highly affect the thermal degradation of the TOCN nanocomposites.

## 4. Conclusions

The TOCN/GO composite films were prepared by incorporating GO and TEMPO-oxidation to investigate the effects of GO nanofillers and oxidation levels. When the amounts of TEMPO and NaBr were increased, the corresponding carboxylate group content was also increased. The well-exfoliated GO nanofillers with various GO contents in the TOCN matrix were verified via XRD. FTIR spectra displayed the increased density of carboxylate groups as the GO content and degree of oxidation increase. Cross-sectional SEM micrographs of TOCN/GO composites represented the morphological changes. For example, compared to neat NFC, they showed thicker and less dense layered structures upon the TEMPO-catalyzed reaction and the inclusion of nanofillers. Nevertheless, TOCN composites at 1.2% and 2% GO loadings exhibited noticeable improvements in the tensile strength and storage modulus, respectively, due to the strong intermolecular cohesiveness between GO sheets and the cellulose matrix. On the contrary, as for 2X TOCN, the increased void volume, caused by intense fibrillation on surfaces, induced a remarkable decrease in both the storage modulus and tensile strength. This corresponds to the results of gas and water vapor permeation properties. The highest oxidation level allowed for a significant increase in the OTR and WVP. In terms of the effect of the various GO contents, the OTR value of 1X TOCN/GO-1.2 was decreased by 82% compared to neat NFC, whereas the WVPs of TOCN/GO composites at different GO loadings were almost identical. The TGA analysis of the thermal properties of neat NFC and TOCN/GO nanocomposites resulted in a promoted thermal degradation of TOCN films owing to an increase in the exposed surface area.

## Figures and Tables

**Table 1:** Carboxylate content with different concentrations of catalysts.

Sample	NaClO (mmol/g)	NaBr (g)	TEMPO (g)	Carboxylate (mmol/g)
neat NFC	-	-	-	0.183
0.5X TOCN	4.46	1.25	0.125	0.280
1X TOCN	4.46	2.5	0.25	0.370
2X TOCN	4.46	5	0.5	0.425

**Table 2:** Percent crystallinity (% *X*c) of the pristine NFC and TOCN/GO nanocomposites obtained by XRD.

Sample	% *X*c
neat NFC	60 (±1.0)
0.5X TOCN	62.3 (±3.5)
1X TOCN/GO-0	62.8 (±2.6)
1X TOCN/GO-0.4	61.7 (±3.4)
1X TOCN/GO-1.2	61.6 (±0.9)
1X TOCN/GO-2.0	62.3 (±5.9)
2X TOCN	61.8 (±2.4)

**Table 3:** Water vapor permeability of neat NFC and TOCN/GO nanocomposite films.

Effect of Graphene Oxide Content		Effect of Degree of Oxidation	
Sample	WVP	Sample	WVP
neat NFC	3.780 (±0.21)	neat NFC	3.780 (±0.21)
1X TOCN/GO-0	0.838 (±0.11)	0.5X TOCN	1.094 (±0.22)
1X TOCN/GO-0.4	0.795 (±0.21)	1X TOCN	0.838 (±0.11)
1X TOCN/GO-1.2	1.650 (±1.24)	2X TOCN	3.288 (±0.90)
1X TOCN/GO-2	1.140 (±0.20)		

Unit of permeability coefficient (P): g·cm/cm^2^·s·Pa (P × 10^−13^).

**Table 4:** OTR values of neat NFC and TOCN/GO nanocomposites at different loadings and different degrees of oxidation.

Effect of Graphene Oxide Content		Effect of Degree of Oxidation	
Sample	OTR	Sample	OTR
neat NFC	993.3 (±81.1)	neat NFC	993.3 (±81.1)
1X TOCN/GO-0	669.7 (±37.3)	0.5X TOCN	176.2 (±9.7)
1X TOCN/GO-0.4	239.8 (±4.3)	1X TOCN	669.7 (±37.3)
1X TOCN/GO-1.2	175.4 (±34.9)	2X TOCN	Not measurable
1X TOCN/GO-2	353.3 (±26.3)		

Unit of oxygen transmission rate: g/m^2^/day.

## Data Availability

Not applicable.
